# WEP: a high-performance analysis pipeline for whole-exome data

**DOI:** 10.1186/1471-2105-14-S7-S11

**Published:** 2013-04-22

**Authors:** Mattia D'Antonio, Paolo D'Onorio De Meo, Daniele Paoletti, Berardino Elmi, Matteo Pallocca, Nico Sanna, Ernesto Picardi, Graziano Pesole, Tiziana Castrignanò

**Affiliations:** 1 Dipartimento di Bioscienze, Biotecnologie e Scienze Farmacologiche, Università degli Studi di Bari, Bari, Italy; 2 CASPUR, Consorzio interuniversitario per le Applicazioni di Supercalcolo per Università e Ricerca, Rome, Italy; 3 Istituto di Biomembrane e Bioenergetica, Consiglio Nazionale delle Ricerche, Bari, Italy; 4 Center of Excellence in Genomics (CEGBA), Bari, Italy; 5 Cineca, Consorzio Interuniversitario di Supercalcolo, Bologna, Italy; 6 Dipartimento di Biotecnologie ed Ematologia, Sapienza Università di Roma, Rome, Italy

## Abstract

**Background:**

The advent of massively parallel sequencing technologies (Next Generation Sequencing, NGS) profoundly modified the landscape of human genetics.

In particular, Whole Exome Sequencing (WES) is the NGS branch that focuses on the exonic regions of the eukaryotic genomes; exomes are ideal to help us understanding high-penetrance allelic variation and its relationship to phenotype. A complete WES analysis involves several steps which need to be suitably designed and arranged into an efficient pipeline.

Managing a NGS analysis pipeline and its huge amount of produced data requires non trivial IT skills and computational power.

**Results:**

Our web resource WEP (Whole-Exome sequencing Pipeline web tool) performs a complete WES pipeline and provides easy access through interface to intermediate and final results. The WEP pipeline is composed of several steps:

1) verification of input integrity and quality checks, read trimming and filtering; 2) gapped alignment; 3) BAM conversion, sorting and indexing; 4) duplicates removal; 5) alignment optimization around insertion/deletion (indel) positions; 6) recalibration of quality scores; 7) single nucleotide and deletion/insertion polymorphism (SNP and DIP) variant calling; 8) variant annotation; 9) result storage into custom databases to allow cross-linking and intersections, statistics and much more. In order to overcome the challenge of managing large amount of data and maximize the biological information extracted from them, our tool restricts the number of final results filtering data by customizable thresholds, facilitating the identification of functionally significant variants. Default threshold values are also provided at the analysis computation completion, tuned with the most common literature work published in recent years.

**Conclusions:**

Through our tool a user can perform the whole analysis without knowing the underlying hardware and software architecture, dealing with both paired and single end data. The interface provides an easy and intuitive access for data submission and a user-friendly web interface for annotated variant visualization.

Non-IT mastered users can access through WEP to the most updated and tested WES algorithms, tuned to maximize the quality of called variants while minimizing artifacts and false positives.

The web tool is available at the following web address: http://www.caspur.it/wep

## Background

Next-generation DNA sequencing has dramatically accelerated biological and biomedical research, allowing the generation of large volumes of data at increasingly lower costs and in shorter times [1-3]. Today the widespread deployment of these technologies has enabled the analysis of genomes, transcriptomes and interactomes [4,5], developing a variety of genome-wide functional assays, such as ChIP-seq [6], RNA-seq [7] and many others.

A widely used application of NGS is Whole Exome Sequencing (WES), a new and efficient approach for studying the genetic basis of human phenotypes [8-10]. The WES technique consists in the selective capture and sequencing of the protein-coding portion of the genome. It has been successfully used to elucidate the genetic causes of many human diseases, starting from single gene disorders and moving on more complex genetic disorders, including complex traits and cancer [11-17].

Through the analysis of WES data it is possible to identify causative variations by comparing cases and controls, also with respect to the variation pattern observed in the normal population. A single experiment can identify several thousands of single nucleotide variants (SNVs) and small insertion or deletion (INDELs) which may contain specific mutations associated to genetic diseases [18,19].

While these achievements have demonstrated the usefulness of exome sequencing compared to whole genome sequencing, which still remains prohibitive for many applications, there are still many bioinformatics challenges that may limit their efficiency/application.

For example, the manipulation and interpretation of the millions of sequences produced by a typical experiment still present significant computational challenges. Analysis of NGS data is troublesome particularly given the short-read lengths and the huge volume of data [20].

Furthermore, sophisticated informatics tools are required [21], and technical skills such as the management and storage of NGS file formats is often impractical and cumbersome [22]. Finally there is no general solution at the present (at least in the author's knowledge) to apply due to the availability of a wide array of data formats, software and analytical tools.

Not less important is the fact that scientists need specific skills in computational biology to mine, analyze and interpret the NGS data.

The development of a streamlined, highly automated pipeline for WES data analysis is one of the possible solutions to address some of these issues. State of the art tools, including GATK, ANNOVAR and SAMTools, can be integrated into a custom pipeline for generating, annotating and analyzing sequence variants.

However, researchers of small biology labs with limited computational resources and experiences should avoid producing once again on their own such an analysis pipeline.

It would be much more efficient to access a web service capable to execute the complete analysis, with simplicity and without requiring any technical informatics skill. This solution thereby would allow the researchers to focus their work on downstream experiments.

In this paper we describe a free web resource, named WEP (Whole Exome sequencing Pipeline web tool): it aims to analyze WES data produced by Illumina platforms [23], which are the most developed and used platforms currently available for WES data production [1,24]. Our web tool automates the execution of an optimized WES pipeline. It is also capable to analyze a user-selected set of exome samples generating tables reporting variant information and their functional annotation.

Through WEP the user is able to quickly perform a WES analysis and identify the biologically significant sequence variations thanks to the use of a simple and intuitive interface. WEP execution does not require any specialized informatics expertise.

Although various analysis pipelines have already been published [25-31] and other solutions based on VirtualBox and Cloud computing are growing in number in recent years [32-35], the authors aren't aware of any web application comparable to WEP at the present moment. None of the other tools available perform a full WES pipeline analysis for free, without installation of codes and other programs on local computers.

## Implementation and methods

WEP has been developed as an ensemble of modules. Its architecture allows each module to run independently, using data stored in MYSQL relational databases. WEP modules are handled by a program completely written in PHP Object Oriented, while mysql libraries are used for database interactions.

WEP has been designed to integrate several in-house developed scripts as well as open source analysis tools into one single pipeline. It handles all required management tasks needed for a cluster distribution of the computation required.

Raw sequence data can be uploaded and results can be viewed using an interactive, web-based graphical user interface (GUI) that has been tested on the main browsers.

The WEP web-based GUI has been written mainly in PHP: Hypertext Preprocessor (PHP) language using HyperText Markup Language (HTML) and JQuery, combined with HTML5 and CSS3 standards, to enable a better user-interaction.

**Figure 1:**
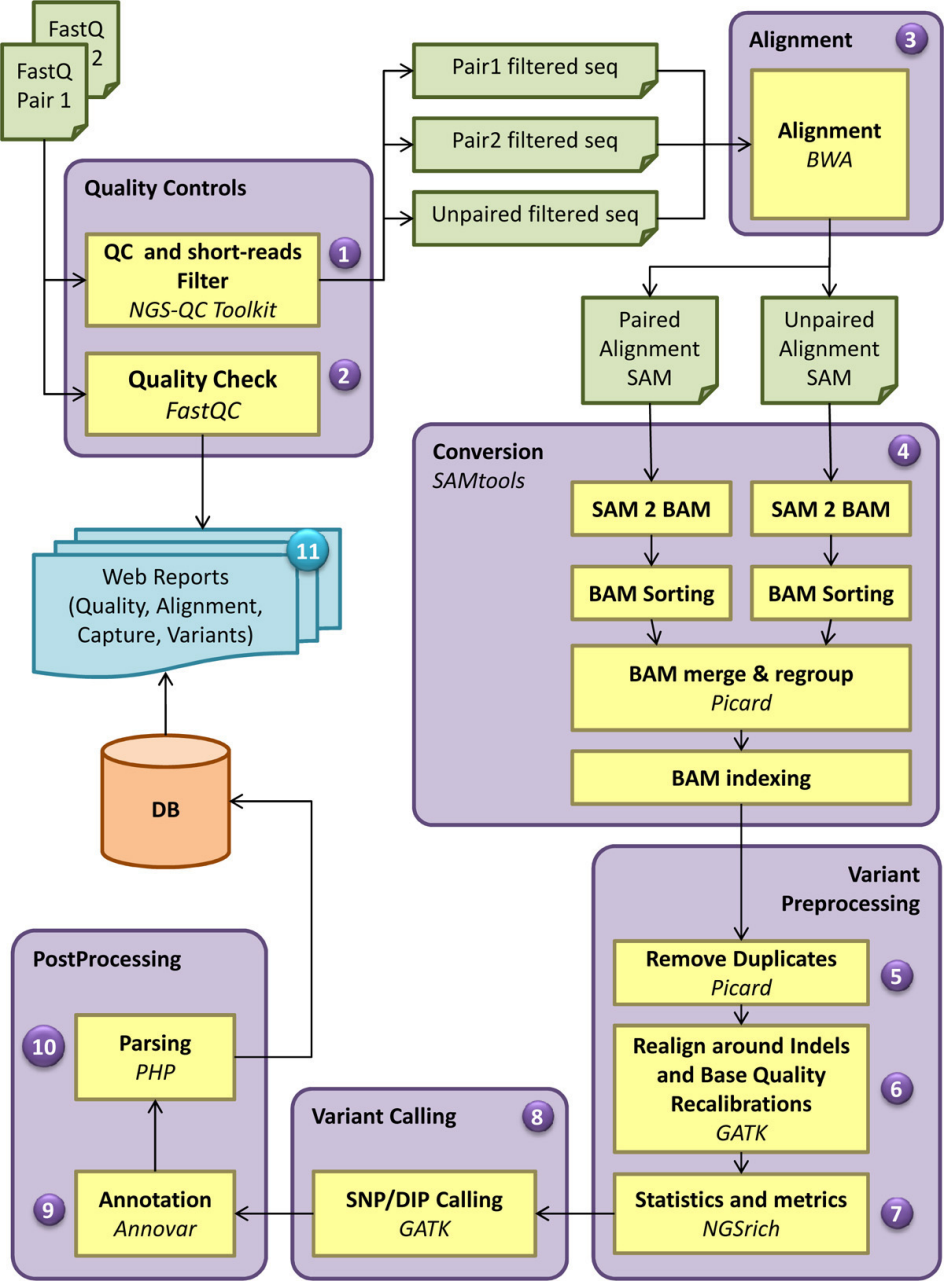
**Bioinformatics analysis workflow of WEP pipeline**. The WEP analysis pipeline consists of 11 major steps, some of which are further divided into sub-components. For each input file a quality control is performed. This step includes both the application of filters and trimmers (1) and the calculation of quality statistics on raw and processed sequences (2). In case of PE reads WEP processes both forward and reverse reads simultaneously and exports the filtered reads in a separate file, keeping the pairing information intact. Unpaired reads passing quality filters are also provided in a different output file. These filtered read are then aligned to their reference genome (3). The two paired files are mapped together (PE alignment), while the unpaired file is aligned individually (SE alignment); for each one is produced a SAM file. Afterwards, WEP executes a conversion step (4) where the resulting SAM files are converted in BAM format, sorted and merged together in a single file. Read groups are assigned and the file is indexed. In the variant preprocessing steps, the duplicates are removed (5), the reads are realigned around indels and the base quality score are recalibrated (6). Furthermore, WEP performs alignment statistics and enrichment target metrics (7). At this point, SNPs and indels are detected (8), several annotation are added to each variant (9) and the results are automatically parsed in optimized databases (10). At the end, WEP collects several information and statistics generated during the pipeline run and generates web pages and reports (11) useful to interpret the performed analysis.

The analysis pipeline includes 11 modules (Figure 1) and performs quality statistics, filtering and trimming of sequence reads, alignment to a reference genome, post alignment analysis with the calculation of mapping rate, statistics and annotation of the detected variants.

WEP takes as input short-read data-sets produced by Illumina sequencing platforms and several standard file formats (FASTQ [36], SRA [37] or BAM [38]) can be accepted. The user can upload these files also in a compressed version to speed up the uploading process (zip, tar, gzip, bz or bz2 compression are admitted); WEP automatically detects the file format and chooses the necessary program to decompress it.

In the following paragraph we describe all the steps of a complete analysis pipeline executed by WEP after data submission (which is detailed in the next paragraph).

1) The first module performs an overall quality check (Quality Control, QC) of uploaded read data. Since this may affect downstream analysis results, an effective QC is critical for a reliable data analysis.

Briefly, for each uploaded FASTQ file WEP carries out a FastQC [39] run generating an HTML web page with detailed QC results in the form of tables and graphs. The user can obtain an overview of some relevant properties of raw reads (such as length, quality scores and base distribution) in order to assess data quality and to discard other low quality reads.

WEP includes also another free application for quality control: the NGS QC Toolkit (NQT) which can directly filter low quality reads after quality check and secondly also removes primer/adaptor sequences [40].

2) The arguably most crucial step of most NGS analysis pipeline is to map short-reads to their source genome. The NGS reads coming from the first step are then aligned to a reference genome. Alignment is usually the most time-consuming operation in a NGS pipeline.

There are many aligners and they differ a lot in performance/efficiency/accuracy [41]. Most alignment algorithms for NGS data are based on either 'hashing' or an effective data compression algorithm the 'Burrows-Wheeler transform' (BWT) [42].

Our pipeline uses BWA [43], based on BWT, a fast and memory-efficient read aligner. BWA is the most common choice for WES alignment [44-46]. It allows gapped alignment, using very little memory. It performs separated alignment on both strands of a paired-end lane, in multi-threaded execution, unifying results in a single mapping file in the Sequence Alignment Map (SAM) format [38].

3) This step is an intermediate phase of the pipeline to pre-process alignment results before further steps.

a) The generated SAM file is converted to the binary Alignment/Map format (BAM), a much more suitable file type because it ensures efficient storing and access to alignment information. The BAM file is indexed to allow the access only to portions of the file without the need to load the whole file.

b) SAMtools [38] are called to order the reads by chromosomal coordinates, to merge the paired and unpaired BAM files of the same sample.

c) the Picard tool [47] is used to tag the read groups (the ID, the library, the sample, the sequence platform).

To enhance the quality of the alignments for more accurate variant detection, the pipeline carries out several "cleanup" procedures (see step 4, 5 and 6 below) before variant calling. These algorithms are also been adopted in the 1000 Genomes Project [48].

4) Early during the rise of NGS, it became apparent that many of the reads from massively parallel sequencing instruments were identical - same sequence, start site and orientation - suggesting that they possibly were PCR artifacts [20]. These duplicates may introduce a bias in estimating variant allele frequencies, and thus it is advisable they are removed prior to the variant calling.

A solution is offered by the Picard suite, which not only applies optimal fragment-based duplicate identification, but marks duplicate reads using the FLAG field rather than removing them from a SAM file. This allows to minimize the experimental artifacts, reducing the number of false calls and improving the accuracy in the search of the variants.

5) The presence of INDELs within the sequences and the gapped alignment read-by-read often lead to false SNP identification towards the end of the aligned reads.

Current mapping algorithms are limited: they align each read independently, without seeing the full context in a region to guide placement of reads, whose first or last few bases overlap an indel tend to have those bases misaligned [48].

These artifacts can be resolved only by examining more sequences in their local context. WEP pipeline carries out a local realignment around INDEL and SNP clusters using the Genome Analysis ToolKit (GATK) realigner [49,50]. It allows to correct misalignments (due to indels) obtaining consensus indels. This module improves the indel calls and reduces false positives in the following step of SNPs calling.

6) The Phred-scaled quality scores indicates the probability of having called a base wrong, formula: Q = -10 log_10(P) [51]. Since it may not accurately reflect the true base-calling error rates [52], necessary for variant calling, we also use a further refinement to quality estimation calculated by the GATK recalibration tool. This application attempts to adjust the base quality scores to be more representative of the underlying data quality. It re-calibrates the base quality values using specific co-variates (as machine cycle and sequence context) and for each unknown base, a re-calibrated quality score is calculated in order to be used for variant calling.

Recalibrating these scores reflects more accurately the empirical probability of mismatches to the reference genome, and by doing so it provides not only more accurate quality scores but also more widely dispersed ones [50].

7) This step of the analysis provides important statistical information in order to assess the quality of data before the search of variants.

SAMtools flagstat is used to generate alignment statistics, i.e. the calculation of the fraction of reads that successfully mapped to the reference, with number and percentages of the read mapped and unmapped, whereas NGSrich [53] provides detailed information about enrichment performance and target coverage, important quality criteria for the evaluation of the exome capture experiment.

8) After all these treatment phases of the read, the basic step of our pipeline is to identify the sites in the sample that statistically differ from the reference genomic sequence. SNPs and DIPs are detected where the reads collectively provide evidence of variation.

As with alignment tools, many algorithms have been developed to identify a high-quality set of variants in NGS projects. The most current variant callers implement base quality and posterior probability calculation to detect variant in NGS data [54].

To carry out this task we included in our pipeline the GATK algorithm, a state of the art program for reliable variant detection [50,55]. It uses a Bayesian statistical model for the calculation of the probability of the genotype, estimating the accuracy of the call with a score of Phred-like quality.

The pipeline is able to detect both SNPs and DIPs simultaneously and the results are reported in a standard Variant Call Format (VCF) file.

9) Before proceeding to the prioritization and filtering of the variants and to rank variations, it is necessary to add several annotation to each detected variant, as the genome position and the functional effect. The WEP pipeline includes the software ANNOVAR [56]. This tool takes advantage of information from different databases and external resources to annotate the variants. The gene annotation, variant function, prediction score of SIFT [57] and PolyPhen2 [58], conservation score of PhyloP [59] and GERP++ [60], the dbSNP [61] ID, the allele frequencies of 1000 Genomes Project [48] and NHLBI-ESP 5400 exomes [62] are only some of the possible annotations that we can retrieve through ANNOVAR tool.

10) At the end of these steps, the pipeline transfers into specifically designed tables (the used DBMS is MySQL Enterprise 5.5) the resulting file, through the use of a specific PHP parser. Therefore the output contains annotated variants with both GATK and ANNOVAR information from previous phases.

11) Once the processed data is stored in the database, WEP generates reports that give end-users sample information to interpret raw data and analysis results.

WEP automatically creates output in one easy-to-navigate HTML page, which provides the project description, QC reports, target coverage and sequencing depth information, descriptions of the annotations, and links to the SNPs and DIPs reports. Each variant is hyperlinked to public databases for the visualization of read alignments and variant calling information at the variant position.

At this point the variants are ready to be analyzed and filtered according to user-defined criteria. A web interface has been developed to allow the user to perform further variant selections, according to the specific biological analysis.

WEP allows to navigate through the results of the experiment, interact with the variants, sort the annotation, export the results as CSV (comma-separates-values) file and much more. The web interface details are shown in the following paragraph.

## Results

### User interface overview

WEP provides an efficient and easy-to-use solution for WES analysis based on NGS data. To achieve this goal, we have implemented our WES tool with a user-friendly web interface. The interactive web application is used to analyze WES data creating specific projects, specifying project parameters, running the required algorithms, and viewing the output of computations by using our High Performance Computing cluster facilities. Through the web interface users can easily view, control and manipulate WES data with few mouse clicks. On the other hand, the interface is also used to deal with heavy computational tasks.

In order to grant security and private access to the user's uploaded data files and results, a login form has been implemented. Registration to the use of WEP is free of charge for academic/research purposes. Data submitted to the WEP interface is private for users, however a public link can be created to share each single result file.

We have developed WEP as modular and extensible as possible, allowing a seamless integration of new data analysis algorithms and visualization features.

**Figure 2:**
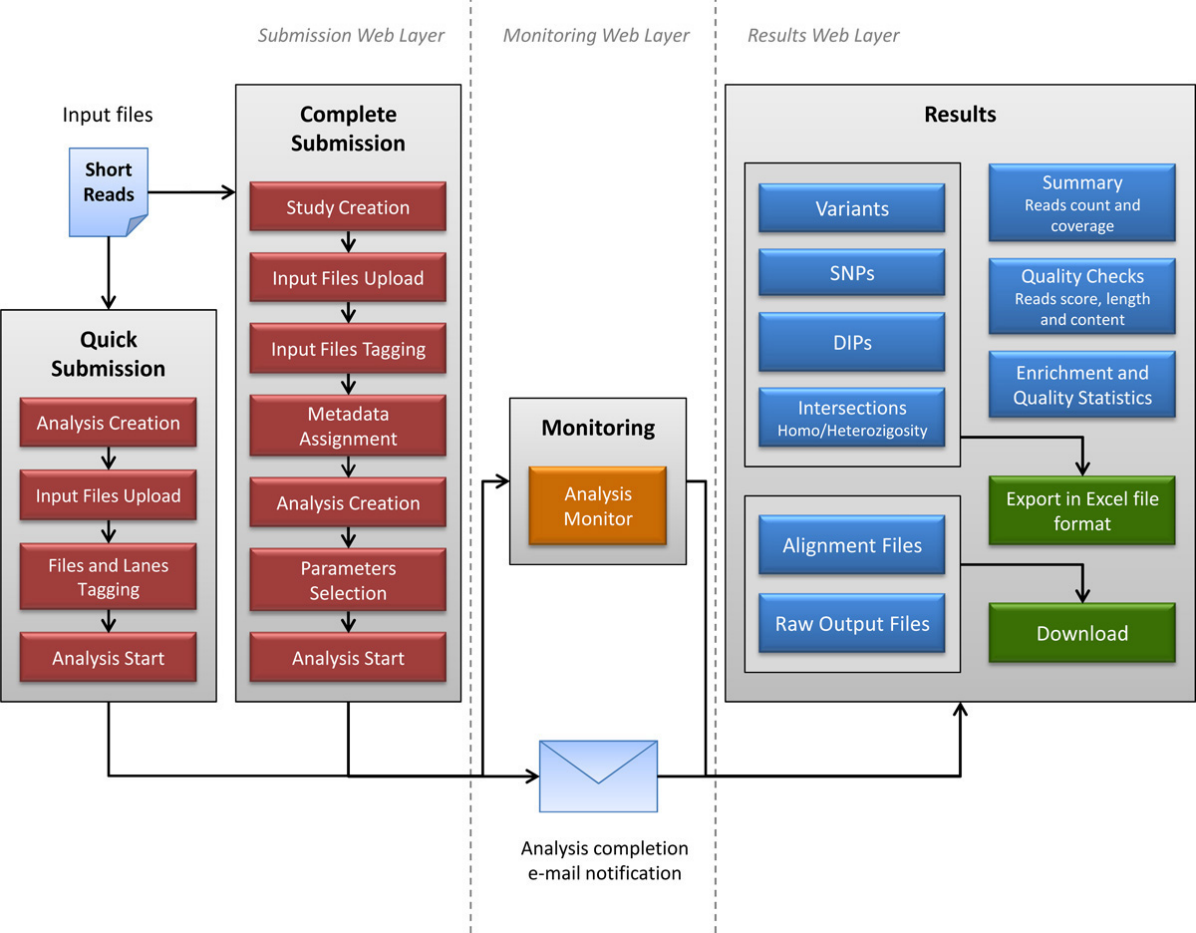
**Workflow of the WEP user interface**. The WEP interface is composed by three main layers: The web submission (on the left) shows the procedures to correctly submit the read input files. Two submission modules are available. The Complete module allows the user to store several information and metadata for each experiment, while in the Quick module the system automatically generates the minimum set of metadata to execute an analysis. The web monitoring (in the center) provides a web page which displays the status of running of WEP pipeline and where the user can visualize and/or download intermediate output results. The web results (on the right) contains all the web pages which show the user all the results obtained from the analysis. These are collected in different sections allowing for an easier viewing. The variant result tables are also exportable in CSV file formats.

The web interface is composed by three main layers: data submission, analysis monitoring and results visualization (Figure 2).

### Submitting the short read sequences

The submission interface workflow is shown on the left part of Figure 2. As described, two submission modules are available: Quick and Complete Submission.

Because of the huge amount of WES data the development of Complete Submission module was needed to introduce a metadata assignment feature. Through this procedure the user can better manage and interpret more in deep WES results.

In the Complete analysis an experiment can be submitted uploading the short reads files and specifying metadata information according to the EBI/ENA data format standards (http://www.ebi.ac.uk/ena/about/sra_submissions).

Taking the SRA data model as our main guideline, we have implemented metadata structure as follows:

Study: information about the a single sequencing project (more analysis can be run in a single study);

Sample: information about the sequenced samples and libraries;

(Actually only human samples can be processed by our analysis architecture.)

Run: information about the platform; associated with study and sample(s);

(Our analysis architecture only admits Illumina runs.)

Analysis: contains the analysis data files, associated with study, sample and run objects.

In the "Complete Submission" panel (as shown in Figure 2), the user can define a new study providing the name and description (Study Creation). After the study creation a form for uploading short reads files is available (Input Files Upload). Then the single or paired end assignment is needed to prepare the analysis (Input Files Tagging). The next phase regards the metadata association to the input files (Metadata Assignment). At this stage the completion of submission is performed by providing the name and description of the analysis (Analysis Creation), by selecting the proper parameters (Parameters Selection) and eventually by launching the analysis (Analysis Start).

The submission process can be also simplified by using the "Quick Submission" module; the first module depicted in Figure 2. In the quick analysis metadata assignment and parameter selection are not required. Study creation and parameter selection are automatically managed by the system. The user needs only to create the analysis, upload input files, tag them and launch the run. This procedure can be useful in case of single input file analysis, very small datasets or lack of metadata information.

Short read datasets have to be uploaded in the standard FASTQ, BAM or SRA format. Also compressed format, such as zip, tar, gzip, bz or bz2 files are allowed to speed up the uploading process. By clicking on the upload button three files at once are loaded and the interface shows several time bars advising the users about the remaining time.

### Analysis execution and monitoring

After submission, a monitoring page providing the list of pipeline steps and the access to the associated output files is available. For each step the running status, the used algorithm and its version, are shown. The running status switches from queued to running, to completed. In case of execution fault, the running status is marked as error. At a completion of each step of the pipeline, all the generated output files are listed and displayed in the monitoring page. In this way the user can visualize or download all the produced raw output files in each step of the pipeline in addition to check the whole analysis progress. For example, when the quality control of the short reads is completed two links to graphical and tabular statistics of qualities are created for both QC tools described in the previous paragraph.

It's also possible to display additional information regarding every single step of user's analysis, e.g. the command line and the estimated running time.

### Gathering output results

After the full analysis completion all raw results are parsed and stored into a dedicated and optimized database. An e-mail containing the link to the available results will be sent from the system to the user email address provided at registration time.

### Summary section

The main results web page shows the summary of the statistics about the whole analysis: the input files, the number of short-reads, total and filtered (after the first step), mapped reads, number of reads after removing duplicates, exome coverage and the number of total variants detected.

Besides, there are additional reports for QC, alignment, enrichment and coverage statistics, and a summary of the resulting variants. The summary includes links to the output files generated by some programs during the execution of the pipeline. This avoids the user to return to the monitoring page of the pipeline to extract such information and have a complete, appealing overview useful for the control of the experiment.

### Quality Checks section

The "Quality checks" section refers to the results of quality control using FastQC [39] and NGS QC Toolkit [40]. The first table shows a summary of the tests executed by FastQC, and gives a quick evaluation of distribution of base quality per read position, deviation from the expected GC content, distribution of fragment sizes, degree of duplication. This section aims to detect possible problems that may be present in the data.

Moreover, NGS QC Toolkit link shows the results of QC displayed both in the form table (number of reads and bases, total and filtered) and in graphs (various QC statistics, as average quality score for each base position, GC content distribution, average Phred quality score distribution).

### Statistics section

The "Statistics" section lead to the alignment and coverage statistics, useful for evaluating data quality and alignment results.

The "mapping statistics" are produced by SAMtools [38]. This section provides a quick summary of the performed alignment, including total number of reads, number of mapped and unmapped reads and the fraction of reads that was successfully paired (only for paired reads).

The "Enrichment performance" section reports the output produced by the NGSrich software [53] for target enrichment evaluation. For each sample a pie chart is provided, giving the fractions of the target regions covered to a particular average depth. Moreover bar plots per chromosome are shown for a quick overview of the coverage across the genome. Further displayed information include the number of genes that are highly or poorly covered (depending on the specific thresholds selected by the user as parameters of the analysis). Eventually a summary table with target-specific coverage information is shown: information about number of reads, mean coverage, fraction of the target region with a particular depth.

### Variants, SNPs and DIPs sections

The "Variants" section shows summary information about detected variants. These are collected in tables containing total variants, SNPs and DIPs. For each of them several information are reported: the total and filtered number of variants, the known variants (such as the variants included in the databases dbSNP and 1000 genomes project).

The default values of the variant filters are: Coverage > 10, ambiguously mapped reads per variant < 5, Phred-scaled consensus quality > 50, variant confidence/consensus quality > 1.5, strand bias Fisher exact test < 60. These filters, in our experience, are suitable to discriminate among true variants and sequencing errors or false positive. However, these values can be changed (with an appropriate form) in function of user's requirements.

By selecting one of the link buttons, contained in the tables, a web page will appear showing the list of genetic variants associated.

**Figure 3:**
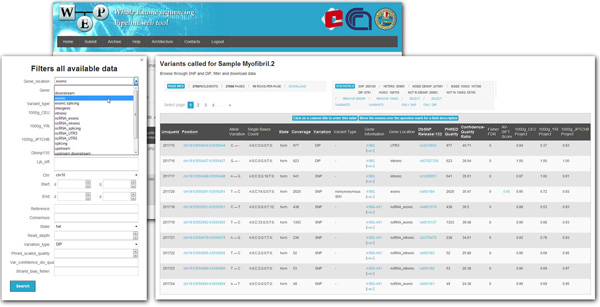
**The filter form on the right shows all the possible filters that can be chosen to select some variants according to the user's requests**. The resulting table on the left, instead, shows the list of genetic variants with all the annotation mined from main public databases (some annotation are also hyperlinked).

This page (Figure 3) provides a simple and convenient interface to browse the results. The output tables can be dynamically sorted, filtered and exported as textual/excel files for offline downstream analyses. A series of annotations mined from the main public databases related to each variant is shown. These information include genomic information (the chromosome and genome position), allelic variation (reference base and identified alternative base), homozygous/heterozygous variant in the sample, type of variation (SNP or DIP), the number and type of bases mapping, variant classifications (synonymous, missense, non-sense, frameshift, etc.), gene name and region (exonic, intronic, intergenic, UTR, etc.), some scores from GATK, dbSNP ID, MAF (minor allele frequency) from 1000 Genomes Project and the SIFT-score.

In this table, some items are also directly linked to main databases: UCSC Genome Browser, Entrez Gene (NCBI), dbSNP (NCBI), 1000 Genomes Project, SIFT (JCVI).

WEP allows to easily filter and remove from the list already known SNPs using information from dbSNP or the 1000 genome project.

### Intersections section

Finally WEP provides an "intersections" section, allowing the user to search for variants shared between samples. The variants are classified as homozygous or heterozygous by the GATK algorithm within each analyzed individual. A boolean search allows to include or exclude each lane. This feature is particularly useful to quickly detect those loci that are either recessive or dominant. For example, in a trio sequencing, with unaffected parents and affected child, it allows to identify the list of loci harboring alleles consistent with the disease inheritance model.

A summary table provides a complete list of genes containing the searched variants. Once again the final table can be downloaded in the same formats as above.

### Data archive

All created studies by each user are listed in the Archive section. From this section it is possible to visualize uploaded files and the analyses executed on them. It is also possible to add new files and run new analyses integrating them to previously created studies. This feature is particularly useful in case of lanes obtained by different experiments and performed at different time or to substitute low qualities lanes. The opportunity to perform several analyses related to the same project allows researchers to easy compare different experimental results.

This section enables the user to increase biological insights on a specific project.

## Conclusions

WEP is a web tool which allows any user to freely submit and perform a complete WES optimized pipeline for Illumina sequenced exome samples. It performs a complete analysis starting from quality controls of submitted short reads produced in single or paired end to SNP/DIP identification and variant annotation.

The GUI, a user-friendly graphic interface, drives the user in each step allowing to browse, search, check, classify and download the identified variants. Furthermore, WEP allows researchers without high-performance computing facilities to build a new study/project and run several analyses. Further samples derived from new experiments can be added to a previously created study in order to increase biological insights.

Output files, status and information about each module of a running analysis are provided. Results can be filtered and managed through the GUI and can be downloaded as CSV standard formats as well. Codes and algorithms are regularly updated and tested.

This web tool performs a complete analysis of whole-exome data speeding up the gathering of results from big data experiments and facilitating the use of WES technologies by laboratories without a specialized bioinformatics staff. Reports generated by our tool are accurate and easy to understand and to use also for non-bioinformatician researchers.

The pipeline executed by WEP is implemented and optimized on a HPC infrastructure to speed up data analysis. This layer is completely hidden to the user, so that the execution of each WES analysis is straightforward and easy.

In order to facilitate the use of the WEP tool, a user help has been developed and is available on the WEP web site, along with a selected FAQ section. The user help includes an application example step-by-step and a video tutorial demonstrating how to use the various options of the application.

Further improvements will be released in future versions of the toolbox.

The WEP tool should be cited in all papers using the results as part of the analysis.

## List of abbreviations used

NGS: Next Generation Sequencing; WES: Whole Exome Sequencing; ChIP-seq: Chromatin Immunoprecipitation sequencing; RNA-seq: RNA sequencing; PCR: Polymerase Chain Reaction; WEP: Whole Exome sequencing Pipeline tool; SAM/BAM: Sequence/Binary Alignment Map; VCF: Variant Call Format; CSV: Comma Separated Values; SRA: Sequence Read Archive; SNV: Single Nucleotide Variants; SNP: Single Nucleotide Polymorphism; DIP/InDel: Deletion Insertion Polymorphism; IT: Information Technology; GUI: Graphical User Interface; QC: Quality Control.

## Competing interests

The authors declare that they have no competing interests.

## Authors' contributions

MD, PDDM, DP, BE, MP developed the web interface along with the whole analysis pipeline. TC, GP supervised the project development. NS acted as technological supervisor. GP, EP acted as scientific supervisors. TC, MD, PDDM, BE, MP wrote the manuscript. All authors read and approved the final manuscript.

## Declarations

The publication costs for this article were funded by the corresponding author's institution.
